# The antioxidant, anti-angiogenic, and anticancer impact of chitosan-coated herniarin-graphene oxide nanoparticles (CHG-NPs)

**DOI:** 10.1016/j.heliyon.2023.e20042

**Published:** 2023-09-09

**Authors:** Louay Mohammed Musa Jasim, Masoud Homayouni Tabrizi, Elham Darabi, Muslem Mohammed Mosa Jaseem

**Affiliations:** a Plasma Physics Research Center, Science and Research Branch, Islamic Azad University, Tehran, Iran; b Department of Biology, Mashhad Branch, Islamic Azad University, Mashhad, Iran

**Keywords:** Chitosan-coated herniarin-graphene oxide nanoparticles (CHG-NPs), Selective cytotoxicity, Antioxidant, Anti-angiogenic, Anti-Lung cancer

## Abstract

**Background:**

Herniarin, a simple coumarin found in chamomile leaf rosettes is known as the oxidative stress protector. In the current study, herniarin was captured into Graphene oxide nanoparticles and coated with chitosan poly-cationic polymer to be used as a novel bio-compatible nano-drug delivery system and investigate its antioxidant, anti-angiogenic and anti-cancer impacts on human lung A549 cancer cells.

**Method:**

The Chitosan-coated Herniarin-Graphene oxide nanoparticles (CHG-NPs) were designed, produced, and characterized utilizing DLS, FESEM, FTIR, and Zeta-potential analysis. The CHG-NPs’ antioxidant activity was analyzed by conducting ABTS and DPPH antioxidant assays. The CHG-NPs’ anti-angiogenic activity was analyzed by CAM assay and verified by measuring VEGF and VEGFR gene expression levels following their increased treatment doses by applying Q-PCR technique. Finally, the CHG-NPs’ cytotoxicity was studied in the human lung A549 cancer cells.

**Result:**

The stable (+27.11 mV) 213.6-nm CHG-NPs significantly inhibited the ABTS/DPPH free radicals and exhibited antioxidant activity. The suppressed angiogenesis process in the CAM vessels was observed by detecting the decreased length/number of the vessels. Moreover, the down-regulated VEGF and VEGFR gene expression of the CAM blood vessels following the increased CHG-NPs treatment doses verified the nanoparticles’ anti-angiogenic potential. Finally, the CHG-NPs significantly exhibited a selective cytotoxic impact on human A549 cancer cells compared with the normal HFF cell line.

**Conclusion:**

The selective cytotoxicity, strong antioxidant activity, and significant anti-angiogenic property of the nano-scaled produced CHG-NPs make it an appropriate anticancer nano-drug delivery system. Therefore, the CHG-NPs have the potential to be used as a selective anti-lung cancer compound.

## 1 Introduction

Lung cancer with 1,796,144 deaths reported in 2020 has assigned about 18% of all cancer deaths worldwide [1]. The cancer metastatic invasion is reported for most patients suffering from lung cancer [2], which is associated by poor life quality [3]. On the other hand, the current common high-risk not-specific cancer therapy strategies for lung cancer do not efficiently overcome its metastatic invasion and uprising mortality. Applying targeted cancer therapy approaches such as bio-compatible drug delivery systems have opened a promising horizon in improving the drug's activity and stability in the blood circulation process [4]. Also, applying herbal-based phytochemicals as the chemical type alternatives have significantly reduced the chemotherapy-mediated undesired side effects.

Nanotechnology has provided appropriate tools for improving therapeutics' bio-accessibility and bio-compatibility. In other words, producing the molecular nano-cage encapsulating the bioactive compounds supplies the drug's gradual release and increases its chemical stability. There are several types of nontoxic biocompatible molecules such as nonionic surfactants, polymers, and phospholipids have been used as the molecular cage for entrapping the bioactive phytochemicals into the nanoemulsions, nanoparticles, and nanoliposomes drug delivery systems, respectively [[5], [6], [7]]. In this regard, there are several phytochemical-loaded drug delivery systems consisting of various types of encapsulators. Huang et al. produced the hybrid gold-Lycopene-loaded nanoemulsions and studied their anticancer impact on colon cancer cell lines [8]. Also, Kavinkumar et al. synthesized the hybrid Graphene oxide-silver nanoparticles and investigated its anticancer impact on the lung A549 cancer cells [9].

Drug delivery systems play an important role in efficiently improving their cargo's bioactivity. This is while the main pharmaceutical activity belongs to the loaded-bioactive compound, which is expected to affect the cancer cell metabolism, suppress its proliferation, and inhibit its metastasis. Cancer cells have to rapidly proliferate and develop into a complex cell colony by supplying the cell-feeding roots or the alternate blood vessels [10].

It has been reported that cancer cells have increased levels of endogenic metabolic-caused Reactive oxygen species (ROS), which makes them vulnerable to exogenic ROS. The increased cancer cell metabolism is controlled by up-regulating the Vascular endothelial growth factor (VEGF) and Vascular endothelial growth factor receptor (VEGFR) gene expression to induce the formation of the required blood vessels for continuous cell survival [11]. Therefore, the anticancer phytochemicals decreasing the cancer cell survival and suppressing the angiogenesis process have the potential to efficiently suppress carcinogenesis.

Herein, the herniarin as a simple coumarin found in chamomile leaf rosettes was selected due to its dual antioxidant/cytotoxic potential [12]. The hydrophobic chemical structure of herniarin limits its bio-accessibility. To overcome the herniarin's low solubility and improve its gradual release, the graphene oxide nanoparticles interacted with herniarin and were coated with chitosan. In the current study, the Chitosan-coated Herniarin-Graphene oxide nanoparticles (CHG-NPs) were produced and characterized to study their antioxidant, anti-angiogenic, and anticancer activity on human A549 cancer cells.

## 2 Materials and methods

### 2.1 Materials

Herniarin (Golexir, Iran), Graphene oxide, ethyl(dimethylaminopropyl)carbodiimide/N-hydroxysuccinimide (EDC/NHS), Chitosan (LMW), 2,2′-azinobis (3-ethylbenzothiazoline-6-sulfonic acid) (ABTS), 2,2-Diphenyl-1-picrylhydrazylradical (DPPH), 3-(4,5-dimethylthiazol-2-yl)-2,5-diphenyltetrazolium bromide (MTT), and dimethylsulfoxide (DMSO) were purchased from Sigma Aldrich. The human lung cancer (A549) and normal (HFF) cell lines were provided by the Pastore Institute of Iran.

### 2.2 CHG-NPs synthesis

The CHG-NPs synthesis was conducted in two steps, producing herniarin-graphene oxide nanoparticles (HG-NPs) and coating HG-NPs with chitosan. To produce HG-NPs, Graphene oxide (10 mg) was dissolved in acetic acid (10 mL, 10% V/V) at the adjusted (pH = 5). The solution was stirred for 1 h at room temperature. Then, 10 mL of herniarin (2 mg/mL) solution was dropwise added to the Graphene oxide solution under continuous stirring conditions and incubated in a shaker incubator for 24 h at 30 °C. The HG-NPs were centrifuged at 10000 rpm for 5 min and rinsed three times to remove impurities. The resulting sediments were lyophilized. The chitosan-coating process was initiated by dissolving HG-NPs in a 1% acetic acid solution. The HG-NPs mixture was homogenized by sonicating at 300 W (8″ On 2” Off) for 60 min. Then, EDC/NHS solution was prepared at a 1:2 ratio and added to the resulting mixture, and stirred for 10 min. Next, the chitosan solution (1% chitosan + 1% acetic acid) was gradually added to the mixture and incubated for 24 h under continuous stirring conditions. The Chitosan-coated HG-NPs (CHG-NPs) were centrifuged at 10000 rpm for 5 min and rinsed three times to remove impurities. The resulting sediments were lyophilized.

### 2.3 CHG-NP characterization

The dynamic light scattering (DLS) analysis was performed to determine the hydrodynamic size dispersion of both HG-NPs and CHG-NPs by volume, number, and intensity (Zetasizer (nanoparticle SZ-100)). To study the morphology and size of the CHG-NPs in dehydrated conditions, Field emission scanning electron microscopy (FESEM) was utilized. In this regard, a drop of CHG-NPs mixture was dried on a piece of clean soft aluminum foil and coated with gold ions for microscopy imaging. The CHG-NPs formation was verified by detecting the chitosan, herniarin, and graphene oxide FTIR spectrums. To this purpose, 2 mg of CHG-NPs were mixed with 200 mg potassium bromide (KBr) and compressed to a thin transparent disc for conducting FTIR analysis (4000–400 cm^−1^ wavenumbers (Resolution = 4 cm^−1^). Finally, the nanoparticles’ stability was evaluated by measuring their surface charge (Zetasizer (nanoparticle SZ-100).

### 2.4 Herniarin loading and releasing efficiency

**Fig. 1:**
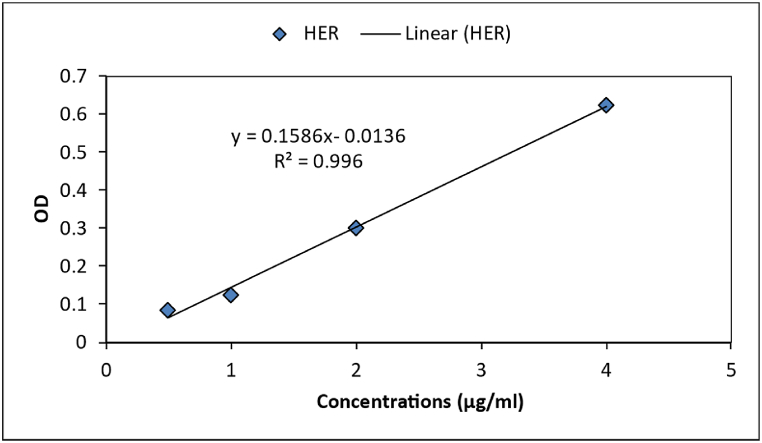
The herniarin standard curve.

The Herniarin loading efficiency on the graphene oxide particles was determined by applying a visible-ultraviolet spectrophotometer (Hutch, USA). Briefly, after the synthesizing process of the nanoparticles, according to the herniarin standard curve (Fig. 1), the drug concentration of the supernatant was measured by recording the herniarin absorbance at 217 nm wavelength.

To measure the drug release rate, CHG-NPs (0.05 g) were placed in 20 ml of phosphate buffer solution in a pre-activated dialysis bag. The bag was suspended in a beaker containing phosphate buffer. Then, the herniarin absorbance was recorded at 217 nm at desired times for the beaker solution. Finally, the resulting graph of the herniarin release was plotted by Excel software.

### 2.5 CHG-NP antioxidant activity

#### 2.5.1 ABTS assay

To measure the CHG-NPs antioxidant activity, its ability in scavenging the ABTS free radicals was measured as previously described. Briefly, the ABTS· radical was activated by potassium persulfate (2.45 mM) and diluted with an equal volume of water. The activated ABTS was stored at room temperature for 14 h in dark conditions. Then, 500 μL of CHG-NPs mixture (62.5, 120, 250, 500, and 1000 μg/mL) was added to 500 μL activated ABTS solution and incubated for 30 min at room temperature in dark conditions. The samples' absorbance was recorded at 743 nm [13]. The ABTS scavenging rate (ASR%) was calculated as the following equation:

ASR% = A control - A sample/ Acontrol × 100

### 2.6 DPPH assay

To prepare the analytical DPPH solution, DPPH (1 mg) was dissolved in ethanol (17 mL). The violet solution was added to a range of CHG-NPs concentrations (62.5, 125, 250, 500, and 1000 μg/mL) and passed 30-min incubation at 37 °C. The samples’ absorbance was recorded at 517 nm wavelength. The DPPH scavenging rate (DSR%) was calculated as the following equation:

DSR% = A control - A sample/ Acontrol × 100

### 2.7 MTT assay

Both A549 lung cancer and normal HFF cells were seeded (5 × 10^3^ cells/cm^2^) and cultured for 24 h in a complete cell culture medium containing (DMEM cell culture media (Gibco), 10% FBS, streptomycin (100 mg/mL) and penicillin (100 U/mL at standard conditions (37 °C, 95%humidity, 5%CO_2_ ventilation). The cultured cells were seeded in 96-well plates at a density of 5 × 10^3^ cells/well and cultured for 24 h at standard conditions. The cultured cells were exposed to a range of CHG-NPs doses (31.2, 62.5, 125, 250, and 500 μg/mL). Following 48-h exposure, the old media was refreshed with fresh media containing MTT (0.5 mg/mL). After a further 3-h incubation at 37 °C, DMSO was added to the wells to dissolve the produced formazan. The concentration of produced formazan was estimated by recording the sample's absorbance at 570 nm as the cells' survival index (Stat fax 2100 plate reader). The cells' viability was measured as the following equation:

Cell survival (%) = (sample absorbance/control absorbance) × 100.

### 2.8 CAM assay

To investigate the effect of nanoparticles on the angiogenesis process, the CAM method was conducted. For this purpose, 40-fertilized eggs were prepared, disinfected, and placed in the incubator for 48 h. Then, a window was created in the eggshell and sealed with sterilized paraffin. The eggs were kept for 6 days at standard conditions (60% humidity, 37 °C, and twice rotation per day). To treat the samples, small sections of gelatin sponge containing a range of CHG-NPs concentrations were prepared using egg white, agar, and antibiotics placed on the chorioallantoic membrane. Then the samples were resealed and transferred to the incubator. The samples' shell window was opened and several images were taken from the exposure area. Images were analyzed with Image J software to estimate the embryos' length/weight and the CAM blood vessels’ length and number [14].

### 2.9 Gene expression profile

For this purpose, Chorioallantoic membranes were separated from each group. Next, the samples' total RNA was extracted by applying an RNA extraction kit (Pars tous, Iran). The extracted RNA was used for the synthesis of the cDNA library utilizing a cDNA synthesis kit (Pars tous, Iran). Primer sets were designed at the exon junction of the VEGF and VEGFR genes by Allel ID6 software (Table 1). The genes’ cDNA was detected by PCR amplification (Bio-Rad CFX96). To measure the fold change of target genes, real‐time PCR was conducted by utilizing a PCR master mix (Qiagen, Hilden, Germany) containing SYBR green dyes. Then, the fold change values were normalized applying a comparative threshold cycle method for GAPDH gene expression.

**Table 1:** The PCR primer sets of target genes (VEGF and VEGFR).

Gene	Forward sequence	Reverse sequence
GAPDH	GCAGGGGGGAGCCAAAAGGGT	TGGGTGCCAGTGATGGCATGG
VEGF	CCTCCGAAACCATGAACTTT	TTCTTTGGTCTGCATTCACATT
VEGFR	CCAGTCAGAGACCCACGTTT	AGTCTTTGCCATCCTGCTGA

### 2.10 Statistical analysis

Data significance levels were determined applying One-way ANOVA statistical analysis (SPSS-20 software). The P-values less than 0.001, 0.01, and 0.05 were defined as statistically meaningful levels illustrated as ***, **, and * index.

## 3 Results

### 3.1 CHG-NP characterization

**Fig. 2:**
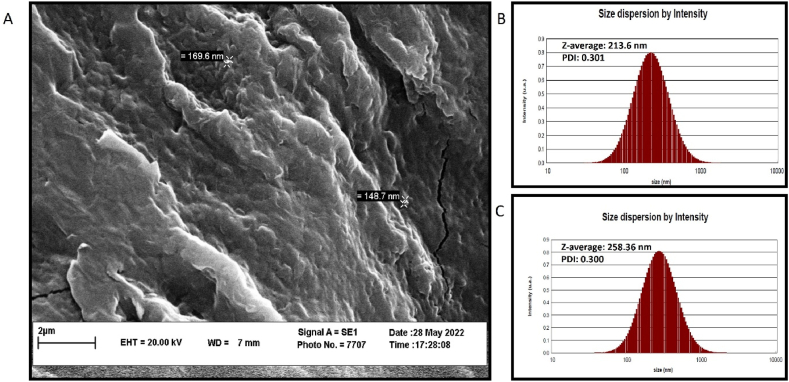
The HG-NPs and CHG-NPs size characterization. A) The FESEM micrograph of the dehydrated CHG-NPs size and morphology. B and C show the hydrodynamic Z-average size of HG-NPs and CHG-NPs, respectively. HG-NPs: Herniarin-Graphene oxide nanoparticles; CHG-NPs: Chitosan-coated herniarin-graphene oxide nanoparticles.

The dehydrated CHG-NPs’ size and morphology were analyzed by FESEM microscopy. The result indicated the smallest 148.7-nm nanoparticles (Fig. 2A). Also, the hydrodynamic size of HG-NPs and CHG-NPs was measured at 213.6 and 258.36, respectively (Fig. 2B and C). To analyze the nanoparticles' dispersity and evaluate the reported size reliability, the Poly-dispersed Index (PDI) was measured. The result showed the PDI index at 0.3 for both HG-NPs and CHG-NPs. The less than 0.7 values for the PDI index are considered the monodispersed nanoparticles and accurate reported size [15].

**Fig. 3:**
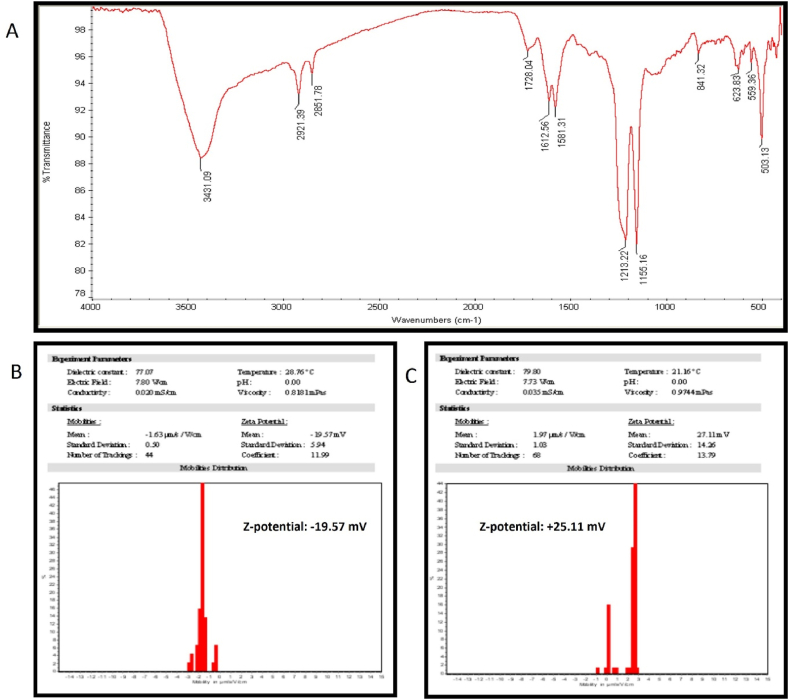
The chemical structure and stability results of the HG-NPs and CHG-NPs. A) represents the FTIR peak pattern of CHG-NPs. B and C indicate the Zeta potential charge og HG-NPs and CHG-NPs, respectively. characterization of HG-NPs and CHG-NPs. HG-NPs: Herniarin-Graphene oxide nanoparticles; CHG-NPs: Chitosan-coated herniarin-graphene oxide nanoparticles.

To verify the CHG-NPs production process, their chemical structure was studied by detecting the functional groups of chitosan, graphene oxide, and herniarin in the FTIR peak pattern of CHG-NPs. As shown in Fig. 3A the band at 1155.16 cm^−1^ (the COC bridge asymmetric stretching) represented both the chitosan and oxadiazole cyclic group of herniarin [[16], [17], [18]]. Also, the O–H and C=O stretching vibrations of the carboxyl group are appeared at 3431.09 and 1728.04 cm^−1^ wavenumbers, respectively [19,20]. The Chitosan coating layer not only increased the HG-NPs size but also modified the HG-NPs surface charge from −19.57 mV to +25.11 mV in CHG-NPs, which can be effective in improving the cellular uptake and overall stability (Figs. 3B and 2C) [21].

### 3.2 Herniarin loading and releasing efficiency

**Fig. 4:**
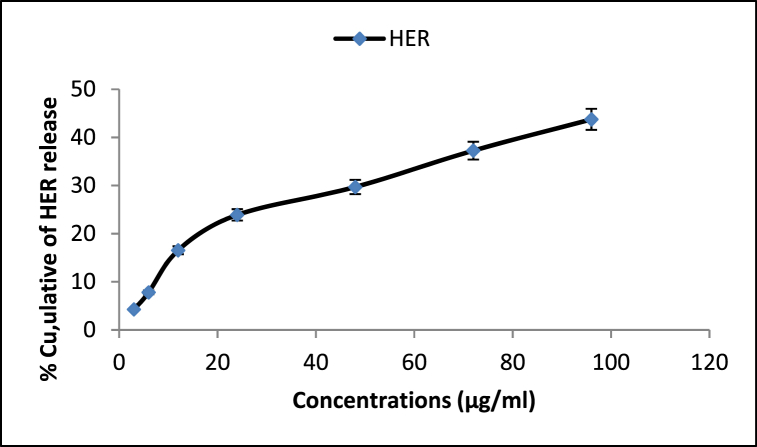
The herniarin releasing rate from the CHG-NPs. CHG-NPs: Chitosan-coated herniarin-graphene oxide nanoparticles.

Examining the amount of drug encapsulation in nanoparticles by spectrophotometer absorption method indirectly indicated the percentage of the encapsulated drug at 93.1%. The drug release rate was estimated at pH 7.4. The results showed a gradual 96-h release rate. In other words, about 43% of the herniarin was released from the CHG-NPs within 96 h. The gradual release can be attributed to the chitosan-coating layer in the CHG-NPs formulation (Fig. 4).

### 3.3 The CHG-NP antioxidant activity

**Fig. 5:**
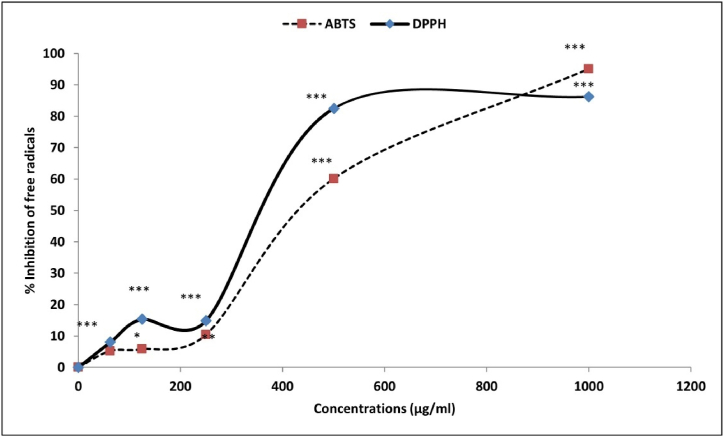
The CHG-NPs activity in scavenging the ABTS and DPPH free radicals. CHG-NPs: Chitosan-coated herniarin-graphene oxide nanoparticles.

Following the increased CHG-NPs concentration, the significant inhibition of both ABTS and DPPH was observed. The CHG-NPs significantly inhibited 50% of both DPPH and ABTS free radicals at < 400 μg/mL and >400 μg/mL concentrations, respectively (Fig. 5). In other words, the nanoparticles illustrated weak antioxidant activity at less than their non-toxic concentrations (200 μg/mL) in both normal and cancer cells, which reflects its potential in accelerating the cancer cell death induction by allowing the ROS accumulation nearby the cancer cells.

### 3.4 The CHG-NP cytotoxicity

**Fig. 6:**
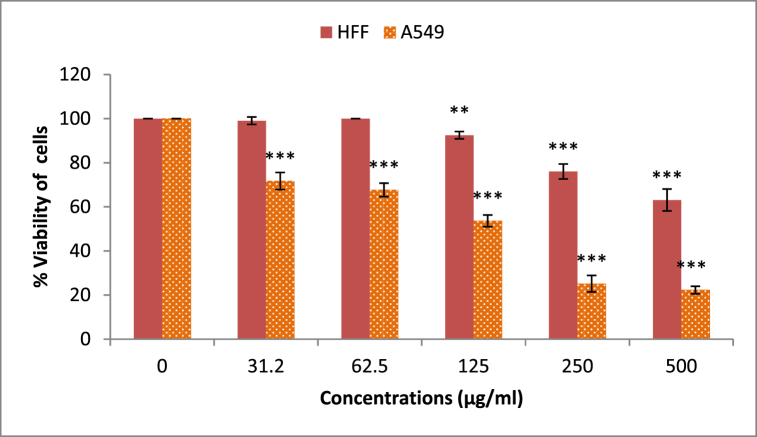
The 48-h treated cell survival in response to the increased CHG-NPs treatment doses. CHG-NPs: Chitosan-coated herniarin-graphene oxide nanoparticles.

The CHG-NPs selectively induce a significant cytotoxic impact on the human lung A549 cancer cells in response to the increased exposure doses compared with normal HFF cells (Fig. 6). The minimum doses of CHG-NPs decreasing 50% of live A549 cells was estimated for the 48-h treated cells at 145.9 μg/mL. This is while the detected IC_50_ doses of CHG-NPs were measured at greater than 500 μg/mL. The CHG-NPs’ cytotoxicity in cancer cells at less than their toxic doses for normal cells indicated the potential of CHG-NPs in inducing selective cytotoxicity.

### 3.5 The CHG-NP anti-angiogenic activity

**Fig. 7:**
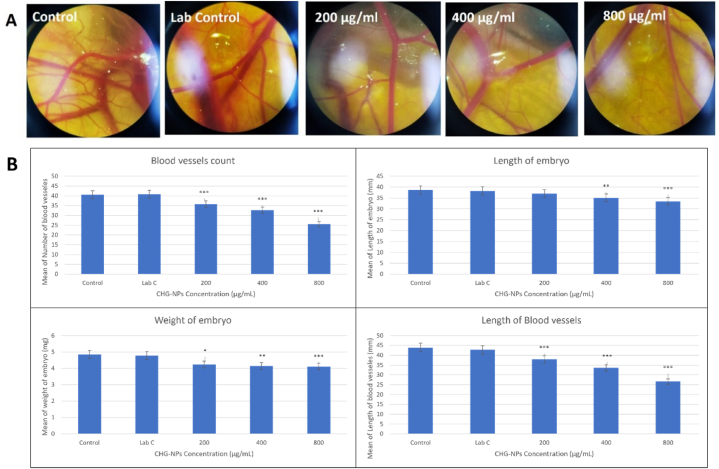
The angiogenesis alteration in the CAM embryo and blood vessels characteristics in response to the increased treatment concentration of CHG-NPs. CHG-NPs: Chitosan-coated herniarin-graphene oxide nanoparticles.

Analyzing both the CAM blood vessels characteristics and quantitative PCR measurement of the CAM gene expression profile (VEGF and VEGFR) exhibited the significant anti-angiogenic activity of the CHG-NPs. The significant decrease in length and count of CAM blood vessels in response to the increased treatment doses of CHG-NPs exhibited their efficient suppressive potential on the CAM angiogenesis process (P-value <0.001) (Fig. 7A and 7B). Moreover, the meaningful suppression of embryo growth was observed by measuring the weight and length of the embryo. A significant association between the decreased weight and length of the embryo with the increased CHG-NP was detected (P-value <0.001) (Fig. 7B).

**Fig. 8:**
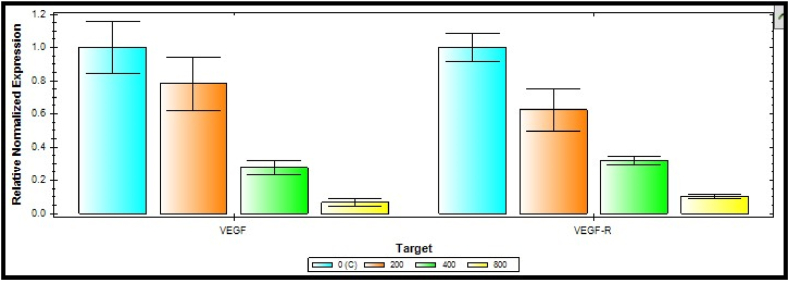
The VEGF and VEGFR gene expression alteration following the increased CHG-NPs treatment concentration in the CAM tissue. CHG-NPs: Chitosan-coated herniarin-graphene oxide nanoparticles.

The CHG-NPs suppressive impact on the angiogenesis process was also verified by detecting the significant decrease in VEGF and VEGFR gene expression (P-value <0.001) at higher doses of CHG-NPs (Fig. 8). Down-regulating the VEGF and VEGFR has the potential to completely suppress angiogenesis process by blocking both the angiogenesis stimulator and its receptor-mediated cellular uptake in the treated cancer cells.

## 4 Discussion

Human lung cancer's poor prognosis and its growing mortality rate are the most challenging issues in the current treatment strategies. Utilizing nano drug delivery systems as the more efficient and selective cancer therapy approaches have opened a promising horizon in efficiently decreasing cancer-caused mortality. In the current study, herniarin molecules were entrapped into graphene oxide nanoparticles and coated with thin polycationic chitosan polymer to improve the bio-accessibility and gradual release of the herniarin delivery system. The chitosan-coated herniarin-graphene oxide nanoparticles (CHG-NPs) were successfully produced and selectively exhibited significant cytotoxic impacts on human lung A549 cancer cells compared with the normal HFF cell line. The CHG-NPs not only exhibited a gradual release of herniarin but also indicated significant antioxidant activity. Finally, the decreased length and count of CAM blood vessels, suppressive growth impact on the chicken embryos, and down-regulated VEGF and VEGFR gene expression of CAM tissue following the increased CHG-NPs treatment doses approved the CHG-NPs anti-angiogenic activity.

The use of medicinal phytochemicals has developed due to their less toxic side effects and multiple bioactivities. Herniarin, of the herbal compounds with therapeutic properties, is Herniarin, a simple coumarin found in chamomile leaf rosettes that has several bioactive effects including antioxidant and anticancer effects. However, their less bio-accessibility and chemical stability in the blood circulation system have limited their bioactivity [22,23]. Nanotechnology has solved the problem by designing nano-drug delivery systems consisting of various types of biocompatible materials such as carbon nanotubes, graphene, and graphene oxide (GO). Among the mentioned carbon-based structures, GO was selected due to its two-dimensional sp2-bonded carbon structure, which makes it enable to easily be functionalized through the π-π interaction with the hydroxyl, carboxylic acid, and epoxy functional groups [24,25].

In the current study, to improve the GO bio-compatibility, chitosan (CS) was selected due to its mucoadhesion, biodegradation, antibacterial activity, low immunogenicity, polyelectrolyte nature, and solubility in various media to coat the herniarin-GO complex [26]. In other words, the chitosan-coating layer not only improves the herniarin stability and its gradual release but also decreases the hemolytic potential of GO and increases its bio-compatibility. The chitosan-coated GO nano-carriers were previously produced by Song et al. [27]. Few studies have reported a chitosan-coated GO nano transporter for delivering bioactive compounds. In this regard, Khoee et al. synthesized chitosan-coated GO mesoporous silica nanoparticles [28]. Also, Chen et al. produced a doxorubicin-loaded GO-CS nanocomposite [29].

The graphene-based nanomaterials (GBNs) such as GO have exhibited several types of unique applications in drug delivery systems due to their acceptable biocompatibility and optimized specific area. The Lower cytotoxicity of GBNs makes them safer for biomedical applications. Moreover, high fracture strength, acceptable thermal stability, electrical conductivity and mobility of GBNs are among their important advantages. GBNs have been used in sensitive biosensors, cell imaging, gene therapy, tissue engineering, and drug delivery systems [30].

Considering the cancer cell survival strategies in supplying the growth energy and in-time migration to other tissues, blocking the angiogenesis activity and selectively reducing the cancer cell survival can efficiently suppress cancer cells’ growth and metastasis [[31], [32], [33]]. Herein, the produced CHG-NPs selectively induced a significant cytotoxic impact on the human lung A549 cancer cells compared with the normal HFF cell line. The selective cytotoxicity of the nanoparticles may be due to the modified up-regulated endocytosis mechanisms in cancer cells compared with normally limited endocytosis in normal cells [34].

On the other hand, the CHG-NPs exhibited a remarkable anti-angiogenesis potential by suppressing the VEGF and VEGFR gene expression. Blocking the angiogenesis process suppresses the cancer cell feeding process and migration ability, which both inhibit cancer carcinogenesis [35]. However, further studies are required to evaluate the anti-angiogenic potential of CHG-NPs on the murine models of human cancers.

## 5 Conclusion

The produced CHG-NPs successfully improved the herniarin bioactivities by protecting its chemical structure and increased its bio-accessibility and cellular uptake based on the GO chemical properties. The nanoparticles selectively induced meaningful cytotoxicity and protected normal cells against the environmental reactive oxygen species as a strong antioxidant compound. Moreover, the CHG-NPs suppressed the angiogenesis process in CAM tissue, which indicated their anti-angiogenic potential. Therefore, the CHG-NPs have the potential to be used as anti-lung cancer and anti-metastatic compound. However, the migration process has to be studied in further cancer cell lines.

## Author contributions

Louay Mohammed Musa Jasim and Muslem Mohammed Mosa Jaseem: Performed the experiments; Contributed reagents, materials, analysis tools or data; Wrote the paper. Masoud Homayouni Tabrizi and Elham Darabi: Conceived and designed the experiments; Analyzed and interpreted the data; Wrote the paper

## Funding

This research was performed at personal expense in the laboratory of Islamic Azad University of Tehran.

## Availability of date and materials

The datasets used and/or analyzed during the current study are available from the corresponding author upon reasonable request.

## Ethics approval and consent to participate

All institutional and national guidelines for the care and use of laboratory animals were followed.

## Consent for publication

Not applicable.

## Additional information

No additional information is available for this paper.

## Declaration of competing interest

The authors declare that they have no known competing financial interests or personal relationships that could have appeared to influence the work reported in this paper.
